# Lateralized modulation of beta-band power in sensorimotor areas during action observation

**DOI:** 10.3389/fnint.2015.00043

**Published:** 2015-06-25

**Authors:** Joachim Lange, Anastasia Pavlidou, Alfons Schnitzler

**Affiliations:** ^1^Medical Faculty, Institute of Clinical Neuroscience, Heinrich Heine-UniversityDüsseldorf, Germany; ^2^Department of Medicine, John A. Burns Medical School and The Queens Medical Center, University of HawaiiHonolulu, HI, USA

**Keywords:** mirror-neuron system, somatotopy, inferior frontal gyrus, premotor cortex, point-light displays

## Abstract

The cortical network for action observation includes areas of the visual cortex and non-visual areas, including areas of the motoric system. Parts of this network are known for their contralateral organization during motion execution, i.e., they predominantly control and respond to movements of the contralateral body side. We were interested whether this lateralized organization was also present during action observation. Human participants viewed point-light displays of human actors, where the actor was facing and moving either to the right or to the left, while participants' neuromagnetic activity was recorded using magnetoencephalography (MEG). We found that right and left facing movements elicited different activity in left and right motoric areas. This lateralization effect was found in two distinct spatio-temporal-spectral clusters: An early lateralization effect in medial sensors at 12–16 Hz and ~276–675 ms after stimulus onset, and a second cluster in more lateral sensors at 22–28 Hz and ~1275–1775 ms. Our results demonstrate that in addition to the known somatotopic organization of parts of the human motoric system, these areas also show a lateralization effect during action observation. Thus, our results indicate that the hemispheric organization of one's own body map known for motion execution extends to the visual observation of others' bodily actions and movements.

## Introduction

The recognition of human movements is an important aspect of social interaction. Observing other individuals provides rich information about their actions, intentions, moods, etc. (see Blake and Shiffrar, 2007 for an overview). The recognition of human movements also shows remarkable characteristics which differentiate the recognition process of human movements from recognition processes of other, non-living objects. For example, human movements and their intrinsic characteristics can be quickly and accurately recognized even if the human body is depicted by only a handful of so-called point-lights attached on the otherwise invisible body (Johansson, 1973).

Imaging studies have revealed a widespread cortical network involved in the perception of human movements. This network includes visual areas, but also higher level cortical areas extending beyond the classical low level visual areas (e.g., Grossman et al., 2000; Saygin et al., 2004; Gilaie-Dotan et al., 2013; Pavlidou et al., 2014b,c). Among these areas is a network known as the mirror neuron system (MNS). The MNS has been first observed in monkeys and is known as a system of neurons which are activated during action execution but also during observation of the action in the absence of active execution (see Rizzolatti and Craighero, 2004 for a review). Most prominent areas of the MNS are the premotor cortex, inferior frontal gyrus and inferior parietal lobule (Rizzolatti and Craighero, 2004). It is still debated whether a MNS analogous to the monkey MNS exists in humans.

An analogous system in the human brain has been supported by single cell recordings in humans (Mukamel et al., 2010) and indirectly by neurophysiological and neuroimaging studies, including EEG and MEG. Several EEG studies reported a suppression of alpha/mu-activity (~8–13 Hz) in sensors over central and motoric areas during action observation (e.g., Muthukumaraswamy et al., 2004; Ulloa and Pineda, 2007; Perry and Bentin, 2009; Frenkel-Toledo et al., 2013).

In addition, MEG studies have demonstrated that action observation leads to a desynchronization of activity in the beta-band (~14–30 Hz). It has been shown that execution, observation and imagination of a movement suppress alpha/beta-band activity, but at different degrees. For example, the suppression of beta-band activity has been shown to be less strong for observation and imagination compared to motion execution (e.g., Schnitzler et al., 1997; Hari et al., 1998; Babiloni et al., 2002). Furthermore, recent studies have demonstrated that beta-band power in sensorimotor areas correlates with the plausibility of the observed action (Pavlidou et al., 2014c).

In addition to the core parts of the MNS observed in monkeys, also other areas in the human brain are relevant for action observation. While these areas may not contain mirror neurons *per se*, they are connected anatomically and/or functionally to the core MNS. In addition, they often show desynchronization of alpha/beta activity in response to action observation, similar to the areas of the MNS. This has led to the notion of an “extended MNS” including, among others, the superior temporal sulcus and sensorimotor areas (Pineda, 2008).

Parts of this extended MNS- mainly the sensorimotor areas—are known for their somatotopic organization. That is, each part of the body is represented in a corresponding area in the sensorimotor cortex. In addition, the somatotopic representation is mainly contralateral, so that sensorimotor area resembles the human body of the contralateral side (Rizzolatti and Luppino, 2001).

While the knowledge of somatotopic organization and hemispheric lateralization is mainly derived from studies on motor execution, studies have shown that the somatotopic representation is also present during action observation. For example, an fMRI study revealed that observation of hand, foot or mouth movements activated different areas in the premotor cortex in accordance with the known somatotopic organization (Buccino et al., 2001).

A largely unresolved experimental question, however, is whether the hemispheric lateralization of sensorimotor areas is also present during action observation. Evidence for a lateralized organization during action observation comes mainly from studies investigating hand movements. For example, EEG and MEG studies reported that observation or imagination of hand movements induces lateralized alpha/beta-band suppression over frontal and central sites (de Lange et al., 2008; Frenkel-Toledo et al., 2013), In the present study, we aimed to investigate whether such lateralized activation is also present during action observation involving the whole body. That is, we studied whether the activation of motoric areas by action observation depends on the observed body side of the actor. We hypothesized that this lateralization would be reflected in differential neuronal oscillatory power, especially in the beta-band. To this end, we employed different human actions depicted as point-light displays and recorded neuromagnetic brain activity while subjects viewed these actions either with the actor facing left or right.

## Methods

The present study uses data from a previously reported study (Pavlidou et al., 2014b,c). While subjects, stimuli and paradigm are thus identical to the previously reported studies, the present study, however, focuses on a different experimental questions and thus data analysis differs from our previous studies.

### Subjects

Twelve subjects (6 male, age: 27.6 ± 2.9 y [mean ± SD]) with normal or corrected-to-normal vision participated in this study after giving written informed consent in accordance to the declaration of Helsinki and the Ethical Committee of the Medical Faculty, Heinrich Heine-University Düsseldorf.

### Stimuli and paradigm

Stimuli and paradigm of the present study were previously reported in detail (Pavlidou et al., 2014b,c). Here we report a concise overview.

Subjects fixated a central red cross for a jittered period (800–1300 ms). Then, additionally, a movie of point-light display (PLD) was presented centrally for 5 cycles (3600–5000 ms). After the PLD presentation, a black screen was presented for a jittered period (0–1000 ms). Finally, response instructions were presented for 2000 ms and subjects were asked to rate the PLD within this period. After the response or after 2000 ms the response text disappeared and the next trial started.

PLDs depicted either a natural action of a human figure or a modified, unnatural (implausible or scrambled) version of the action and subjects were asked to rate the plausibility of the action. All stimuli were presented in random order. Since in the present study data analysis will focus only on a subset of the natural actions, we will describe only natural actions in detail here. For a detailed description of the unnatural actions see Pavlidou et al. (2014b,c).

**Figure 1:**
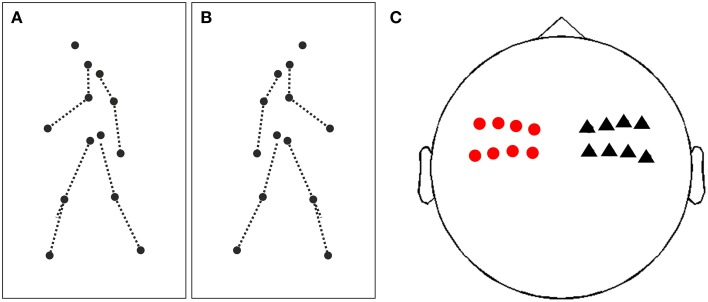
**Illustration of stimuli and sensors of interest. (A)** Illustration of a single frame of a point-light display (PLD) facing and walking to the left. PLDs are represented by 13 black dots, dashed lines are only for illustration and not present in the movies. **(B)** Same as **(A)**, but now a PLD walking and facing to the right. **(C)** Illustration of sensors of interest for time-frequency analysis and subsequent analysis of lateralization effect. Red circles represent 8 sensors in the left hemisphere, black triangles 8 sensors in the right hemisphere, covering bilateral (pre)motor areas.

PLDs were generated by recording the movements of human actors with 13 sensors attached to their main joints (head, shoulders, elbows, wrists, hips, knees, and feet) using a motion tracking system [MotionStar; Ascension Technology, Burlington, VT; (Lange and Lappe, 2007)]. Movements consisted of natural actions (e.g., walking, skipping, throwing) viewed either from the left, right or frontal view. All translatory motion was subtracted offline so that the PLDs were walking in place. Since in the present study we were interested in the putative lateralization of neuronal activity in response to left and right movements, we used only the stimuli facing left or right (see Figures 1A,B and Movies 1, 2 for examples).

### MEG recordings and data analysis

While subjects viewed the stimuli, we recorded neuromagnetic activity with a 306-channel whole head MEG system (Elekta Neuromag Oy, Helsinki, Fnland) with a sampling rate of 1000 Hz. Vertical and horizontal electrooculograms were recorded simultaneously for offline artifact rejection.

Data were offline analyzed using custom-made Matlab (The Mathworks, Natick, Massachusetts, USA) scripts and the Matlab-based open source toolboxes FieldTrip (Oostenveld et al., 2011) (http://fieldtrip.fcdonders.nl).

Continuously recorded MEG data were offline epoched in trials starting with the onset of the fixation cross and ending with the presentation of the response instructions. All trials were semi-automatically and visually inspected for artifacts. Artifacts caused by muscle activity, eye movements or SQUID jumps were removed semi-automatically using a z-score based algorithm implemented in FieldTrip. In a nutshell, data was filtered in a frequency band known to be sensitive for muscular (110–140 Hz) or ocular (1–14 Hz) artifact. Next, *z*-values for each channel were computed for each time point, resulting in a time course representing standardized deviations from the mean of all channels. Artifacts were identified and removed by applying a threshold and cutting out segments exceeding this threshold. The threshold was individually set for each subject and manually chosen, depending on individual noise levels and data quality (Lange et al., 2013). Excessively noisy channels were removed and reconstructed by an interpolation of neighboring channels. Additionally, power line noise was removed from the segmented data by using a band-stop filter encompassing the 50, 100, and 150 Hz components.

Spectral power for the frequency band 4–40 Hz was computed for each sensor separately by applying a discrete Fourier Transformation on sliding time windows of 500 ms length, moved in steps of 20 ms. Data segments were first multiplied with Hanning window, resulting in an effective smoothing of ±2 Hz. The two orthogonal channels of each gradiometer pair were combined by summing the power of the two channels.

For each subject, spectral power was averaged separately over trials depicting a PLD facing to the left or to the right, respectively. Next, we chose two sets of a priori defined sensors covering left and right (pre)motor areas (Figure 1C; eight left sensors: “MEG0212+0213,” “MEG0222+0223,” “MEG0232+0233,” “MEG0242+0243,” “MEG0412+0413.” “MEG0422+0423,” “MEG0432+0433,” “MEG0442+0443”; eight right sensors: “MEG1112+1113,” “MEG1122+1123,” “MEG1132+133,” “MEG1142+MEG1143,” “MEG1312+1313,” “MEG1322+1323,” “MEG1332+1333,” “MEG1342+1343”). In each sensor set, we pooled for each time-frequency pixel spectral power for right and left facing PLDs across all sensors of interest and then computed a lateralization index (LI) for each time-frequency pixel in each sensor set (right or left sensors). The LI was defined as the differences of spectral power between right and left PLDs divided by their variance (i.e., equivalent to an independent sample *t*-test). This approach provided for each subject and each sensor set (right or left) a time-frequency map of LI.

To evaluate whether right and left hemispheres showed a differential activation by PLD, we finally statistically compared the LI for right and left sensor sets across subjects using a non-parametric randomization test (Maris and Oostenveld, 2007).

To this end, we compared the LI for right and left sensor sets by a time-frequency-wise dependent samples *t*-test. This approach led to a time-frequency map of *t*-values. To correct for multiple comparisons, we applied a cluster-based randomization approach (Maris and Oostenveld, 2007). To this end, *t*-values were thresholded at a value of *t* = 1.96 (i.e., *p* = 0.05), and neighboring time-frequency-points exceeding this threshold were clustered. Values within a cluster were summed, giving our cluster-level test statistic. We generated a randomization distribution by randomly exchanging the t-maps of a random subset of subjects. The cluster-statistics were recomputed for these new group-level pooled t-maps. By repeating this step 1000 times, a randomization distribution of cluster-level test-statistics was computed and the test statistics of the observed clusters were compared with this randomization distribution (for details see Lange et al., 2011, 2013). This non-parametric approach avoids assumptions about underlying distributions, implements a random effect analysis, and corrects for multiple comparisons across time and frequency (Maris and Oostenveld, 2007).

In summary, our approach results in a multiple-comparison corrected time-frequency map of values that indicate how strongly activation in response to left and right PLD differs between hemispheres.

**Figure 2:**
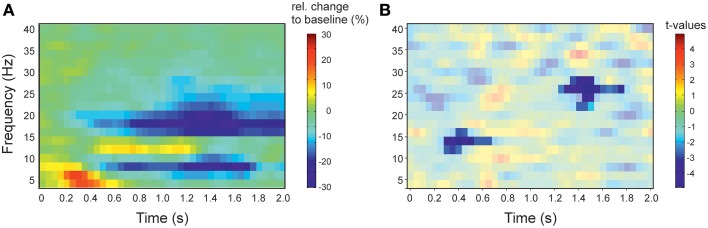
**Spectral profile of stimulus induced activity and results of the contrast of left vs. right sensors. (A)** Time-frequency representation of the activity in response to action observation. Spectral activity was averaged across left and right sensors. Activity is presented as relative change to baseline (−500 to −100 ms). Red colors indicate increased activity relative to baseline, blue colors decrease of activity. **(B)** Time-frequency representation of the statistical comparison of the lateralization index (right vs. left facing actions) in right vs. left sensors of interest. *t* = 0 denotes the onset of stimulation. Red colors indicate greater lateralization index in right sensors compared to left sensors, blue colors indicate a smaller lateralization index. Results are masked to highlight significant clusters. Colorbar applies to the significant (non-masked) pixels.

In addition, we performed *post-hoc* ANOVAs and *t*-tests on the significant clusters found in the above mentioned analysis. To this end, we averaged spectral power over significant time-frequency pixels (as defined in Figure 2) separately for each condition (left or right PLD) and sensor set (left or right hemisphere). Averaged power values were log-transformed and then forwarded to a 2 × 2 ANOVA with the factors PLD direction (left/right) and hemisphere (left/right).

To test an influence of body posture on lateralization of spectral power, we extracted for each action sequences of maximally and minimally informative body postures. Maximally informative postures were defined as postures which show the largest difference between a rightward oriented posture and its mirrored leftward counterpart. Hence, minimally informative postures show the smallest difference. The respective body postures were determined by subtracting for each point the horizontal positions of left and right postures and then summing up the differences of individual points. We averaged spectral power at the time point of maximal/minimal difference ± 100 ms. Averaged power values were log-transformed and then forwarded to a 2 × 2 × 2 ANOVA with the factors PLD direction (left/right), hemisphere (left/right), and body posture (maximal/minimal).

## Results

We found a significant negative cluster between 275 and 675 ms and 12–16 Hz (*p* = 0.045) and second negative cluster between 1225 and 1775 ms and 22–28 Hz (*p* = 0.004), i.e., the difference between left and right facing point-light displays (PLD) showed the strongest lateralization effect in the beta-band (Figure 2).

To further elucidate the lateralization effect, we performed a *post-hoc* analysis on these significant time-frequency clusters. To this end, we performed a 2 × 2 ANOVA with the factors direction (left/right) and hemisphere (left/right). For the early cluster (275–675 ms), the ANOVA revealed no significant main effects (factor direction: *F* = 0.03, *p* = 0.87, factor hemisphere: *F* = 0.01, *p* = 0.93) but a highly significant interaction effect (*F* = 8.6, *p* = 0.008). For the late cluster (1225–1775 ms), the ANOVA revealed no significant main effects (factor direction: *F* = 2.31, *p* > 0.14, factor hemisphere: *F* = 0.37, *p* > 0.55) but a highly significant interaction effect (*F* = 16.1, *p* ≤ 0.001).

**Figure 3:**
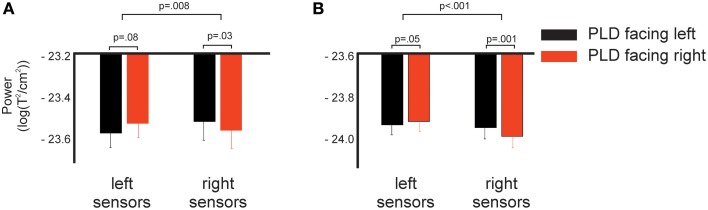
**Spectral power in response to left and right facing actions for left and right sensors as shown in Figure 1C. (A)** Log-transformed spectral power averaged across time-frequency points of the early significant cluster shown in Figure 2 (275–675 ms, 12–16 Hz). **(B)** Same as **(A)**, but now for the late cluster (1225–1775 ms, 22–28 Hz). *p*-values indicate results of *post-hoc t*-tests (lower row) and interaction effect of the 2 × 2 ANOVA (upper row, see Methods and Results for details).

For the early cluster, *post-hoc t*-tests revealed a strong trend toward significance (*p* = 0.08) for the comparison in the left hemisphere and a significant difference for the comparison in right sensors (*p* = 0.03). That is, in right sensors, PLD facing to the left elicited stronger power in the beta-band compared to PLD facing to the right and in left sensors, PLD facing to the right elicited stronger power in the beta-band compared to PLD facing to the left (Figure 3A).

For the late cluster, *t*-tests revealed a very strong trend toward significance (*p* = 0.05) in left sensors and a highly significant (*p* < 0.001) difference in right sensors, i.e., in left sensors PLD facing to the right elicited stronger power in the beta-band compared to PLD facing to the left while in right sensors the opposite pattern was found: PLD facing to the left elicited stronger power in the beta-band than PLD facing to the right (Figure 3B).

**Figure 4:**
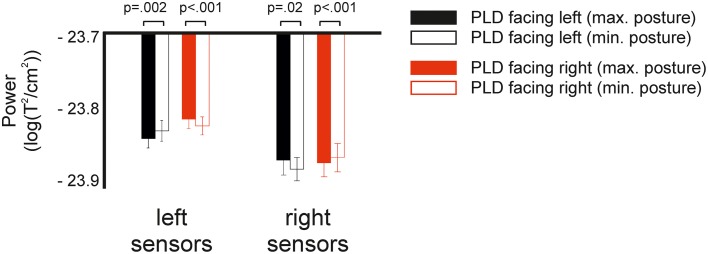
**Spectral power in response to left and right facing actions at specific body postures**. Fully colored bars show power in response to body postures which maximally differentiate between left and right facing bodies. White bars with colored outline show power in response to body postures which minimally differentiate. *P*-values indicate results of *post-hoc t*-tests between maximally and minimally differentiating postures.

To test whether the lateralization effect depended on body postures, we performed an additional 2 × 2 × 2 ANOVA with the factors direction (left/right), hemisphere (left/right), and body posture (maximal/minimal) (see Methods for details). The ANOVA revealed a significant main effect of the factors direction (*F* = 8.1, *p* = 0.02) and hemisphere (*F* = 15.2, *p* = 0.002). In addition, we found a significant interaction for direction × hemisphere (*F* = 5.1, *p* = 0.04) and a highly significant effect for the Three-Way interaction direction × hemisphere × body posture (*F* = 30.1, *p* < 0.001). *Post-hoc t*-tests revealed a significant difference between maximal and minimal body postures for all four pairwise comparisons (*p* < 0.02, Figure 4).

**Figure 5:**
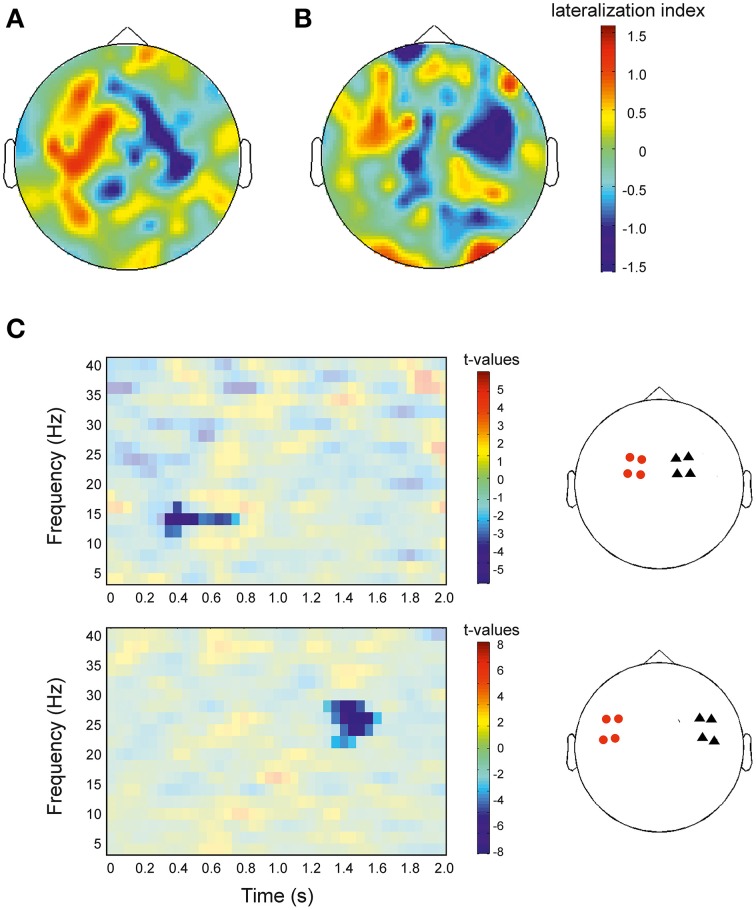
**Topographical representation of the lateralization index and statistical comparison of lateralization index for distinct sensor sets. (A)** Illustration for the early significant cluster shown in Figure 2 (275–675 ms, 12–16 Hz) and Figure 3A. **(B)** Same as **(A)**, but now for the late cluster (1225–1775 ms, 22–28 Hz; Figures 2, 3B). Red colors indicate greater power for right facing actions compared to left facing actions (positive lateralization index), blue colors indicate smaller power for right vs. left facing actions. Colorbars apply to both plots. **(C)** Left panel: Same analysis and representation as in Figure 2B, but now separately for the more medial sensors (upper row) and for the more lateral sensors (lower row). *t* = 0 denotes the onset of stimulation. Red colors indicate greater lateralization index in right sensors compared to left sensors, blue colors indicate a smaller lateralization index. Results are masked to highlight significant clusters. Colorbar applies to the significant (non-masked) pixels. Right panel: Red circles indicate four sensors in the left hemisphere and black triangles indicate four sensors in the right hemisphere used for statistical analysis shown in the left panel.

In accordance with the results shown in Figures 2, 3, the topographical representation of the lateralization effect showed a positive LI in the left hemisphere and a negative LI in the right hemisphere (Figure 5). Visual inspection indicated that both, early and late effects were mostly pronounced in motoric areas, with the early significant effect being more pronounced to medial sites (Figure 5A) compared to the late significant effect (Figure 5B). To test this observation, we repeated the analysis of Figure 2, but now separately for the eight lateral and the eight medial sensors. In medial sensors, only the early cluster reached significance (*p* = 0.044), while in lateral sensors, only the late cluster reached significance (*p* = 0.001; Figure 5C). This analysis, thus, confirmed that the early significant effect was more pronounced to medial sites while the late significant effect was more pronounced in lateral sites.

## Discussion

Suppression of neuronal activity, especially in the alpha- and beta-band, in motoric systems during action observation has been interpreted as an involvement of the respective areas in the process of action observation (e.g., Babiloni et al., 2002; Pineda, 2008; Kilner et al., 2009; Frenkel-Toledo et al., 2013). Here, we found that human actions with the actor facing either to the right or to the left elicited lateralized activity in motoric areas: Right and left areas of the motoric system showed significantly different activation in response to right and left facing actions. This lateralization effect was found at two distinct time periods and spectral clusters: An early significant cluster between 275 and 675 ms and 12–16 Hz (*p* < 0.05) and second cluster between 1225 and 1775 ms and 22–28 Hz (*p* < 0.01). The topographical representation of both effects showed a spatial overlap covering presumably frontal, premotor and motor areas, with the early effect showing a stronger focus toward medial sites and the late effect a more lateralized location.

It is known from studies on action execution that suppression of beta-band power is mainly found in sensorimotor areas contralateral to the movement (Gross et al., 2005). Thus, one also might expect stronger suppression of beta band power in areas contralateral to the body side the actor displays toward the observer. In other words, viewing the actor facing to the right and thus mainly observing the right body side would be expected to elicit stronger suppression of beta-band power in the left hemisphere and vice versa. *Post-hoc* analysis of our data revealed, however, that beta-band power in left sensors was lowest for left facing actions and in right sensors for right facing actions. A potential reason for this effect might be that our point-light actors always displayed both body sides. That is, although for each action there was always one body side directed to the observer, there was no occlusion of remote point-lights when they moved behind the body. In addition, there was no specific task regarding the body side. Since action direction and body side was irrelevant for the task, remote body sides might be included in the recognition process to produce a “whole-body” representation of the action. This might lead to overcompensation effects when the observer tried to embody the remote, ipsilateral body side or imagine the movement of the remote body side.

In a study by Kilner et al. (2009) subjects viewed whole body movements of human actors while EEG activity was recorded. The actor always faced toward the observer while performing an action either with the right or left arm. Similar to the results of our study, Kilner et al. reported that beta band suppression in sensorimotor areas was strongest in sensors ipsilateral to the arm performing the action, i.e., movements of the left arm induced strongest suppression in the left hemisphere and vice versa for right arms. The authors argued that the observed pattern was driven mainly by the side of the screen on which the observed movement occurred and not by the hand that was observed moving. An influence of the side of the screen, however, cannot explain our results since actions were always presented centrally. Our results therefore argue that while the hemifield in which the action is presented certainly plays a role for the strength of the beta-band suppression, there is an additional effect of body side.

de Lange et al. (2008) studied beta-band suppression in sensorimotor areas during motor imagery of hand movements. The authors reported that the duration of beta-band suppression was correlated with the difficulty of the imagery task: The more complex a task or process, the longer beta-band suppression lasts. Observing actions might thus initially induce similar beta-band suppression in both hemispheres independent of the body side viewed. The duration of the beta-band suppression, however, might depend on the body side processed, leading to shorter beta-band suppression in left sensors if the right body side is viewed in comparison to viewing the right body side and vice versa for right sensors. The different duration of beta-band suppression might thus explain the results reported in our study (Figure 3).

In a recent study, Pavlidou at al. (2014b) analyzed the beta-band activity in response to normal (plausible) and biomechanically implausible human actions (using the same dataset as in the present study). The authors reported that beta band suppression was significantly stronger for implausible than plausible actions. The authors argued that the stronger suppression might result from stronger matching of incoming visual information to stored representations of the actions in (pre)motor areas. Thus, rather than reflecting an activation of the MNS *per se*, beta-band suppression might reflect the complexity of a task (Pavlidou et al., 2014a).

We found that the contrast between left and right facing actors was stronger in right than in left hemispheres. This finding is in line with results from studies on action observation and motor imagery. For example, Kilner et al. (2009) reported that the difference of beta-band power between observing left and right arm movements was stronger in right than in left hemispheres. In addition, de Lange et al. (2008) studied imagery of left and right hand movements. The authors reported for the contrast left vs. right hand a stronger suppression of beta-band power in right than in left hemispheres. Similarly, previous studies reported that the left parietal and premotor cortices are equally involved in imagined movements of left and right hands, while the right parietal and premotor cortices are preferentially involved in imagined movements of the contralateral left hand (Parsons et al., 1998; De Lange et al., 2006; Stinear et al., 2006).

In addition, we found that the contrast between left and right facing actors was stronger at the later time cluster. The timing of this later effect is in line with other studies investigating modulations of beta-band power in response to action observation and imagery. For example, Kilner et al. (2009) reported significant differences in beta-band power between observing left and right hand movements to peak at 1670 ms. Pavlidou et al. (2014b) studied the contrast between plausible and implausible actions and reported that the difference in sensorimotor beta-band power was found at 2400–2650 ms. In addition, de Lange et al. (2008) reported differences in beta-band power between imagery of left and right hand movements around 1500 ms.

Early and late cluster differ also with regard of the frequency for which the lateralization effect was found. The early cluster was found to be significant between 12 and 16 Hz. Typically, this frequency band might be assigned to the beta-band (13–30 Hz). While the frequency bands between 6 and 10 Hz and 16–30 Hz show a suppression of activity in response to action observation, the frequency band between 12 and 16 Hz shows an increase of activity (Figure 2A). The spectral profile of the activation pattern in response to action observation, thus, argues for a separate frequency band between ~12 and 16 Hz (Figure 2A). There is evidence for a functional distinction of the alpha-frequency band in a lower and and an upper alpha-band (Klimesch et al., 1997; Klimesch, 1999). In sensorimotor areas, the lower band (8–10 Hz) has been suggested to be somatotopically non-specific while a somatotopically specific oscillation is characteristically found in the upper alpha (10–13 Hz) frequency band (Pfurtscheller et al., 2000). The distinct profile of the 12–16 Hz band underlying the early cluster argues thus that the early cluster might be functionally separate from the late cluster which is clearly located in the beta-band. The early cluster might be related to the somatotopically specific upper alpha band (Pfurtscheller et al., 2000).

In addition to their temporal and spectral profile, the early and the late cluster seem to differ also with regard to their cortical origin. While both clusters spatially overlap, the late cluster clearly extends to more lateral sides than the early cluster (Figure 5). We can only speculate about the cortical sources. The early, more medial cluster might reflect activity in sensorimotor or (pre)motor areas. These areas are known to be somatotopically organized (Buccino et al., 2001). Therefore, the potential spectral overlap with the upper alpha-band, which is thought to reflect somatotopically specific activity (Pfurtscheller et al., 2000), provides further evidence for the sensorimotor areas. The late cluster might origin from inferior frontal areas (Nishitani and Hari, 2000) or premotoric areas. Sensorimotor and premotor areas and inferior frontal gyrus are known to be involved in the process of action observation (Nishitani and Hari, 2000; Rizzolatti and Craighero, 2004; Molenberghs et al., 2012). Our results imply that in addition to their somatoptopical organization, these areas show a lateralized organization with right and left hemispheres being differently activated by left or right facing actions.

In conclusion, we demonstrate that parts of the human motoric system show a lateralization effect with regard to action observation. That is, left and right hemispheres are activated differently by actions for which the actor was facing to the right or to the left. These effects are found for two sensor arrays, presumably covering sensorimotor areas, (pre)motor areas and/or inferior frontal areas. The lateralization effects are found in the beta-band, with the lateralization effect being more strongly pronounced at ~1500 ms after stimulus onset in putative inferior frontal areas. These results demonstrate that during action observation parts of the human MNS show in addition to the known somatotopic organization a lateralization.

### Conflict of interest statement

The authors declare that the research was conducted in the absence of any commercial or financial relationships that could be construed as a potential conflict of interest.

## References

[B1] Babiloni C. Babiloni F. Carducci F. Cincotti F. Cocozza G. Del Percio C.. (2002). Human cortical electroencephalography (EEG) rhythms during the observation of simple aimless movements: a high-resolution EEG study. Neuroimage 17, 559–572. 10.1006/nimg.2002.1192 12377134

[B2] Blake R. Shiffrar M. (2007). Perception of human motion. Annu. Rev. Psychol. 58, 47–73. 10.1146/annurev.psych.57.102904.190152 16903802

[B3] Buccino G. Binkofski F. Fink G. R. Fadiga L. Fogassi L. Gallese V.. (2001). Action observation activates premotor and parietal areas in a somatotopic manner: an fMRI study. Eur. J. Neurosci. 13, 400–404. 10.1111/j.1460-9568.2001.01385.x 11168545

[B4] De Lange F. P. Helmich R. C. Toni I. (2006). Posture influences motor imagery: an fMRI study. Neuroimage 33, 609–617. 10.1016/j.neuroimage.2006.07.017 16959501

[B5] de Lange F. P. Jensen O. Bauer M. Toni I. (2008). Interactions between posterior gamma and frontal alpha/beta oscillations during imagined actions. Front. Hum. Neurosci. 2:7. 10.3389/neuro.09.007.2008 18958208 PMC2572199

[B6] Frenkel-Toledo S. Bentin S. Perry A. Liebermann D. G. Soroker N. (2013). Dynamics of the EEG power in the frequency and spatial domains during observation and execution of manual movements. Brain Res. 1509, 43–57. 10.1016/j.brainres.2013.03.004 23500633

[B7] Gilaie-Dotan S. Kanai R. Bahrami B. Rees G. Saygin A. P. (2013). Neuroanatomical correlates of biological motion detection. Neuropsychologia 51, 457–463. 10.1016/j.neuropsychologia.2012.11.027 23211992 PMC3611598

[B8] Gross J. Pollok B. Dirks M. Timmermann L. Butz M. Schnitzler A. (2005). Task-dependent oscillations during unimanual and bimanual movements in the human primary motor cortex and SMA studied with magnetoencephalography. Neuroimage 26, 91–98. 10.1016/j.neuroimage.2005.01.025 15862209

[B9] Grossman E. Donnelly M. Price R. Pickens D. Morgan V. Neighbor G.. (2000). Brain areas involved in perception of biological motion. J. Cogn. Neurosci. 12, 711–720. 10.1162/089892900562417 11054914

[B10] Hari R. Forss N. Avikainen S. Kirveskari E. Salenius S. Rizzolatti G. (1998). Activation of human primary motor cortex during action observation: a neuromagnetic study. Proc. Natl. Acad. Sci. U.S.A. 95, 15061–15065. 10.1073/pnas.95.25.15061 9844015 PMC24575

[B11] Johansson G. (1973). Visual perception of biological motion and a model for its analysis. Percept. Psychophys. 14, 201–211. 10.3758/BF0321237815820512

[B12] Kilner J. M. Marchant J. L. Frith C. D. (2009). Relationship between activity in human primary motor cortex during action observation and the mirror neuron system. PLoS ONE 4:e4925. 10.1371/journal.pone.0004925 19290052 PMC2654140

[B13] Klimesch W. (1999). EEG alpha and theta oscillations reflect cognitive and memory performance: a review and analysis. Brain Res. Rev. 29, 169–195. 10.1016/S0165-0173(98)00056-3 10209231

[B14] Klimesch W. Doppelmayr M. Pachinger T. Russegger H. (1997). Event-related desynchronisation in the alpha band and the processing of semantic information. Brain Res. Cogn. Brain Res. 6, 83–94. 10.1016/S0926-6410(97)00018-9 9450602

[B15] Lange J. Christian N. Schnitzler A. (2013). Audio–visual congruency alters power and coherence of oscillatory activity within and between cortical areas. Neuroimage 79, 111–120. 10.1016/j.neuroimage.2013.04.064 23644355

[B16] Lange J. Lappe M. (2007). The role of spatial and temporal information in biological motion perception. Adv. Cogn. Psychol. 3, 419–428. 10.2478/v10053-008-0006-3 20517525 PMC2864996

[B17] Lange J. Oostenveld R. Fries P. (2011). Perception of the touch-induced visual double-flash illusion correlates with changes of rhythmic neuronal activity in human visual and somatosensory areas. Neuroimage 54, 1395–1405. 10.1016/j.neuroimage.2010.09.031 20854915

[B18] Maris E. Oostenveld R. (2007). Nonparametric statistical testing of EEG- and MEG-data. J. Neurosci. Methods 164, 177–190. 10.1016/j.jneumeth.2007.03.024 17517438

[B19] Molenberghs P. Cunnington R. Mattingley J. B. (2012). Brain regions with mirror properties: a meta-analysis of 125 human fMRI studies. Neurosci. Biobehav. Rev. 36, 341–349. 10.1016/j.neubiorev.2011.07.004 21782846

[B20] Mukamel R. Ekstrom A. D. Kaplan J. Iacoboni M. Fried I. (2010). Single-neuron responses in humans during execution and observation of actions. Curr. Biol. 20, 750–756. 10.1016/j.cub.2010.02.045 20381353 PMC2904852

[B21] Muthukumaraswamy S. D. Johnson B. W. McNair N. A. (2004). Mu rhythm modulation during observation of anobject-directed grasp. Brain Res. Cogn. Brain Res. 19, 195–201. 10.1016/j.cogbrainres.2003.12.001 15019715

[B22] Nishitani N. Hari R. (2000). Temporal dynamics of cortical representation for action. Proc. Natl. Acad. Sci. U.S.A. 97, 913–918. 10.1073/pnas.97.2.913 10639179 PMC15430

[B23] Oostenveld R. Fries P. Maris E. Schoffelen J.-M. (2011). FieldTrip: open source software for advanced analysis of MEG, EEG, and invasive electrophysiological data. Comput. Intell. Neurosci. 2011:e156869. 10.1155/2011/156869 21253357 PMC3021840

[B24] Parsons L. M. Gabrieli J. D. Phelps E. A. Gazzaniga M. S. (1998). Cerebrally lateralized mental representations of hand shape and movement. J. Neurosci. 18, 6539–6548. 9698341 10.1523/JNEUROSCI.18-16-06539.1998 PMC6793195

[B25] Pavlidou A. Schnitzler A. Lange J. (2014a). Beta oscillations and their functional role in movement perception. Transl. Neurosci. 5, 286–292. 10.2478/s13380-014-0236-424993895

[B26] Pavlidou A. Schnitzler A. Lange J. (2014b). Distinct spatio-temporal profiles of beta-oscillations within visual and sensorimotor areas during action recognition as revealed by MEG. Cortex 54, 106–116. 10.1016/j.cortex.2014.02.007 24657479

[B27] Pavlidou A. Schnitzler A. Lange J. (2014c). Interactions between visual and motor areas during the recognition of plausible actions as revealed by magnetoencephalography. Hum. Brain Mapp. 35, 581–592. 10.1002/hbm.22207 23117670 PMC6869263

[B28] Perry A. Bentin S. (2009). Mirror activity in the human brain while observing hand movements: a comparison between EEG desynchronization in the mu-range and previous fMRI results. Brain Res. 1282, 126–132. 10.1016/j.brainres.2009.05.059 19500557

[B29] Pfurtscheller G. Neuper C. Krausz G. (2000). Functional dissociation of lower and upper frequency mu rhythms in relation to voluntary limb movement. Clin. Neurophysiol. 111, 1873–1879. 10.1016/S1388-2457(00)00428-4 11018505

[B30] Pineda J. A. (2008). Sensorimotor cortex as a critical component of an ‘extended’ mirror neuron system: does it solve the development, correspondence, and control problems in mirroring? Behav. Brain Funct. 4:47. 10.1186/1744-9081-4-47 18928566 PMC2577683

[B31] Rizzolatti G. Craighero L. (2004). The mirror-neuron system. Annu. Rev. Neurosci. 27, 169–192. 10.1146/annurev.neuro.27.070203.144230 15217330

[B32] Rizzolatti G. Luppino G. (2001). The cortical motor system. Neuron 31, 889–901. 10.1016/S0896-6273(01)00423-8 11580891

[B33] Saygin A. P. Wilson S. M. Hagler D. J. Bates E. Sereno M. I. (2004). Point-light biological motion perception activates human premotor cortex. J. Neurosci. 24, 6181–6188. 10.1523/JNEUROSCI.0504-04.2004 15240810 PMC6729669

[B34] Schnitzler A. Salenius S. Salmelin R. Jousmäki V. Hari R. (1997). Involvement of primary motor cortex in motor imagery: a neuromagnetic study. Neuroimage 6, 201–208. 10.1006/nimg.1997.0286 9344824

[B35] Stinear C. M. Fleming M. K. Byblow W. D. (2006). Lateralization of unimanual and bimanual motor imagery. Brain Res. 1095, 139–147. 10.1016/j.brainres.2006.04.008 16713588

[B36] Ulloa E. R. Pineda J. A. (2007). Recognition of point-light biological motion: mu rhythms and mirror neuron activity. Behav. Brain Res. 183, 188–194. 10.1016/j.bbr.2007.06.007 17658625

